# Transitions from biomedical to recovery-oriented practices in mental health: a scoping review to explore the role of Internet-based interventions

**DOI:** 10.1186/s12913-017-2176-5

**Published:** 2017-04-07

**Authors:** Monica Strand, Deede Gammon, Cornelia M. Ruland

**Affiliations:** 1 Centre for Shared Decision Making and Collaborative Care Research, Oslo University Hospital, P.O. Box 4950, Nydalen, Oslo, 0424 Norway; 2 Department of Psychiatry Blakstad, Division of Mental Health and Addiction, Vestre Viken Hospital Trust, Asker, Norway; 3 Faculty of Medicine, University of Oslo, Oslo, Norway; 4 Norwegian Centre for Integrated Care and Telemedicine, University Hospital in North Norway, Tromsø, Norway

**Keywords:** Internet, Secure email, Recovery-oriented care, Service user involvement, Patient-physician relationship, Long-term mental illness, Values-based research and practices

## Abstract

**Background:**

The Internet is transforming mental health care services by increasing access to, and potentially improving the quality of, care. Internet-based interventions in mental health can potentially play a role in transitions from biomedical to recovery-oriented research and practices, but an overview of what this may entail, current work, and issues that need addressing, is lacking. The objective of this study is to describe Internet-based recovery-oriented interventions (referred to as e-recovery) and current research, and to identify gaps and issues relevant to advancing recovery research and practices through opportunities provided by the Internet.

**Methods:**

Five iterative stages of a scoping review framework were followed in searching and analyzing the literature. A recovery framework with four domains and 16 themes was used to deductively code intervention characteristics according to their support for recovery-oriented practices. Only Internet-based interventions used in conjunction with ongoing care were included.

**Results:**

Twenty studies describing six e-recovery interventions were identified and originated in Australia, Finland, the Netherlands, Norway and USA. The domain supporting personal recovery was most clearly reflected in interventions, whereas the last three domains, i.e., promoting citizenship, organizational commitment and working relationship were less evident. Support for the formulation and follow-up of personal goals and preferences, and in accessing peer-support, were the characteristics shared by most interventions. Three of the six studies that employed a comparison group used randomization, and none presented definitive findings. None used recovery-oriented frameworks or specific recovery outcome measures. Four of the interventions were specific to a diagnosis.

**Conclusion:**

Research about how technologies might aid in illuminating and shaping recovery processes is in its formative stages. We recommend that future e-recovery research and innovation attend to four dimensions: evidence-supported interventions, new knowledge about personal recovery, values-based approaches and Internet as a facilitator for organizational transformation. The incremental changes facilitated by e-recovery may help propel a shift in mental health care toward recovery-oriented practices.

## Background

Internet-based interventions are transforming mental health care services by increasing access to, and potentially improving the quality of, care [1]. While there is substantial evidence for online therapies targeting mild or moderate conditions such as depression and anxiety [2–6], less is known about Internet interventions for people with more complex, long-term mental health problems. Several studies show that Internet-based self-management interventions are feasible and acceptable to service users but that more research is needed in designing such interventions and evaluating their effectiveness [7–12]. Two reviews have a specific recovery-oriented perspective related to individuals with bipolar disorder [7] and serious mental illness [12].

The current study specifically examines the role that Internet-based interventions may be playing in the field of personal recovery for those with long-term mental health problems as recently called for [7]. Literature suggests that Internet can facilitate empowerment processes on a personal, interpersonal, group and citizen level [13] exemplified by increased access to social support and reduced stigma [14], power transitions between providers and service users [15] and person-centered services by allowing flexible and individually tailored services in homes and daily lives [16, 17]. At the same time, Internet-related technologies such as self-help apps, social media and virtual reality are developing so rapidly that there is a need to proactively identify and assess ways to exploit their benefits and limit their pitfalls. To aid in this, a number of theories and approaches in the technical and social sciences seem well aligned with the field of personal recovery and can offer some guidance [17–21]. Nevertheless, a systematic overview of Internet-based interventions relative to recovery research is needed to more specifically identify gaps and issues worth addressing, also through alliances with other fields.

### Recovery

Personal recovery, hereafter referred to as recovery, has been defined as; “a way of living a satisfying, hopeful, and contributing life even with any limitations caused by illness” [22]. While not dismissing the important role that biomedical interventions can play for some individuals, recovery challenges biomedical approaches to recovery that prioritize symptom reduction in ways that can inadvertently undermine progress towards a life worth living [23, 24]. Proponents call for a fundamental shift away from the often paternalistic and pacifying nature of biomedical approaches, towards partnerships that acknowledge and support the decisive role that service-users and families play in defining and enacting their own recovery and wellbeing [23, 25]. This personal approach to recovery emerged from within consumer and civil rights movements, and increasingly guides reforms in mental health in English-speaking countries [26]. The approach is person-centered, values-based and is also increasingly reflected in the broader domain of chronic care research and practice [27]. Recovery-oriented interventions recommended in the 2014 NICE guidelines for psychosis as described by van der Krieke et al. [28] include Individual Wellness Recovery Action Planning (WRAP) [29], Illness management and recovery (IMR) [30] and Individual Placement Support (IPS) [31]. However, the complex, multiple and interacting components of such interventions are challenging to implement [32–35], especially compared with pharmacological interventions, despite the latter’s higher risk of adverse events [28].

The holistic and multifaceted nature of recovery-oriented interventions has prompted efforts to operationalize components, also by examining links to related research such as positive psychology, wellbeing, strengths-based approaches and self-management [32, 36, 37]. In their systematic review and narrative synthesis of 97 papers from 13 countries, Leamy and co-workers [38] propose that personal recovery can be conceptualized as a process comprised of five dimensions: connectedness to others and the community; hope and optimism about the future; identity building beyond being a patient and towards a positive sense of identity without stigma; meaning in life; and empowerment, summarized in the acronym CHIME. The CHIME framework has been validated by service users and provides a theoretical base for clinical and research purposes [38].

While CHIME offers a conceptualization of personal recovery Le Boutillier et al. [26] offer a framework for characterizing recovery practices. They identified 16 dominant themes grouped in four practice domains that characterize recovery-oriented practice: (1) promoting citizenship, e.g., supporting the experience of wider entitlements of citizenship such as service user rights, social inclusion and meaningful occupation; (2) organizational commitment, e.g., giving primacy to the needs of people rather than those of services; (3) supporting personally defined recovery, e.g., informed choice, peer support, focus on strengths, and a holistic approach; and (4) working relationships, e.g., a therapeutic relationship that encourages partnership and promotes hope. In this paper, Internet-based interventions that support the four practice domains are referred to as e-recovery. Along with evolving conceptual frameworks for recovery-oriented practices, efforts are underway to identify meaningful outcome measures across cultures and contexts and to identify active ingredients of recovery, for whom, by whom, under what conditions [39–42].

## Objectives

The aim of the current study is to provide an overview of e-recovery interventions and research as an aid in identifying gaps and issues relevant to advancing recovery research and practices through opportunities provided by the Internet. We limit our focus to interventions that are integrated into ongoing care for persons with long term mental health problems in need of long-term mental health support. Arguably, Internet-based interventions that augment existing models of care, in contrast with self-help apps that are detached from ordinary care, are in need of particular attention, also in light of the organizational challenges that can be expected during implementation.

More specifically we addressed the following questions:

1. What characterizes e-recovery interventions (i.e., aims, target groups, settings and modules)?
2. How is recovery supported through the e-recovery interventions?
3. What aims, methods, outcome measures and results are described in the studies and where do they originate?
4. What facilitators and barriers are described in implementing the e-recovery interventions?

The study is to our knowledge the first attempt to describe in detail what may characterize recovery-oriented Internet-based interventions in conjunction with ordinary care.

## Method

Scoping reviews are suitable for charting new territory between areas of research and in identifying issues worth further attention [43]. Scoping studies are defined as “[…] *a form of knowledge synthesis that addresses an exploratory research question aimed at mapping key concepts, types of evidence, and gaps in research related to a defined area or field by systematically searching, selecting, and synthesizing existing knowledge*” [44]. In scoping studies researchers can incorporate a range of study designs and address questions beyond those related to intervention effectiveness, and generate findings that can complement the findings of clinical trials [45]. However, the quality of included studies is not assessed, nor are findings synthesized [43].

This scoping review followed the framework proposed by Arksey and O’Malley [43] and further enhanced by Levac et al. [45] and was accordingly conducted in five stages also guided by Peters et al. [46]. The stages progress in an iterative process, requiring researchers to engage reflexively in each stage, repeating and revising each step whenever necessary to ensure that the literature and research questions are adequately illuminated [43].

### Stage 1

In stage 1, our initial research questions were defined. Although these remained more or less the same in foci and objectives, they were adjusted somewhat during the research process to result in those listed above under Objectives.

### Stage 2

In stage 2, relevant studies were identified based on the research questions and purpose of the study. Due to our interest in mapping research-based literature, we chose to exclude gray literature. Systematic searches for articles published from January 2004 to May 2015 were carried out in the following electronic databases: MEDLINE, PsycINFO, EMBASE and Cinahl. In light of the rapidity of technological developments, we judged this period to be sufficient. Each database was searched using the database thesaurus and the key word/free text method. Searches included the following terms and varying synonyms and related concepts, alone and in various combinations: ‘mental illness’, ‘Internet interventions’, ‘recovery’, and ‘mental health service user–provider interaction’. The term recovery was supplemented with related concepts such as ‘positive psychology’, ‘empowerment’, ‘strengths’, ‘well-being’ and ‘self-management’. All types of study design were included in the search strategy, which was restricted to articles in English or Scandinavian languages and published in peer-reviewed journals. We also searched the reference lists of included studies and relevant conferences for pertinent publications.

### Stage 3

**Fig. 1:**
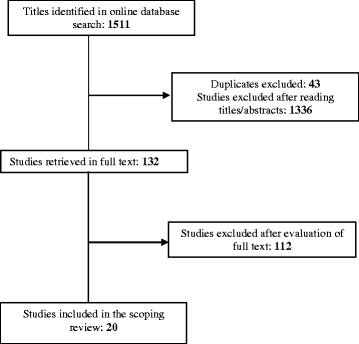
Study selection process. A total of 1511 articles were identified as potentially relevant for the study. Based on reading titles and abstracts, 132 articles were considered relevant for further assessment by reading them in full text. Then, based on the inclusion and the exclusion criteria, 20 articles were included in the scoping review

Stage 3 entailed the process of study selection, illustrated in Fig. 1. In accordance with scoping study principles, selection was an iterative process of reviewing abstracts, refining the research strategy, and developing and revising inclusion and exclusion criteria [43, 45]. We identified a total of 1511 articles (Fig. 1) based on inclusion and exclusion criteria as described in Table 1.

**Table 1:** Inclusion and exclusion criteria

Inclusion criteria:	Exclusion criteria:
Target group	
Individuals with long term mental health problems Individuals > 18 years old	Individuals with substance abuse problems only
Intervention	
Internet based; web-pages and applications for computers, smart phones and tablets	Solely phone calls, text messaging, voice response or videoconference
Part of ongoing treatment and care	Stand-alone interventions
Support for recovery-oriented practices as described by the interventions’ aims and components	Solely support for • self-guidance • monitoring • managing care
Study design	
All study designs, including development reports	Editorials/comments, reviews
Type of publication	
Published in peer-reviewed journals	Gray literature
Language	
English or Scandinavian	Non-English and non-Scandinavian

After removal of duplicates and irrelevant studies based on the titles and abstracts (e.g., not about Internet interventions, not recovery-oriented or wrong target group), 132 potentially relevant studies remained.

All of the potentially relevant studies were read in full text by the first author. The second author independently read a random sample of 20% and additionally all of the articles where the first author had questions about inclusion and exclusion. Disagreements about study selection were resolved by discussion.

### Stage 4

Stage 4 entailed charting the data of the included studies by extracting and coding in Excel each included article according to each of the following variables: intervention’s country of origin, aims, theoretical concepts, target group, settings, modules, study aims, design and methods, outcome, measures and results, implementation issues and the four practice domains and underlying 16 themes of recovery-oriented practice as described by Le Boutillier et al. [26]. The process of extracting the data was done by the first and second authors separately and then together to resolve any discrepancies in coding through consensus. When minor adjustments were made in the data charting form, the included articles and extracted data were reviewed again to ensure correspondence [43, 45].

### Stage 5

The fifth and final stage of the scoping review entailed collating, summarizing and reporting the results [45]. Inspired by a semantic-level, thematic analysis approach [47], the four practice domains and 16 underlying themes served as a point of departure for collating and summarizing the interventions’ characteristics according to their support for recovery-oriented practice. During this processes we made memos that served as a basis for answering the research questions, as well as topics deserving attention in the discussion of our findings. Any discrepancies in interpretations of article content or the predefined domains and underlying themes were resolved through discussion [48]. Again, this is in line with the iterative nature of all the stages in scoping reviews [43, 45].

## Results

A total of 20 articles describing six interventions were included in the scoping review. The interventions originated in Australia, Finland, the Netherlands, Norway and USA.

### Characteristics of the e-recovery interventions

The modules are described in Table 2, together with a description of aim, target group and setting for each intervention.

**Table 2:** Characteristics of the e-recovery interventions

Intervention and origin	Aim	Target group and setting	Modules
CommonGround, USA [49, 53, 54, 81–83]	Recovery and shared decision-making	For persons 18 years or older with severe and persistent mental illness at an outpatient psychiatric medication clinic.	A program consisting of a peer-to-peer workshop and training of providers in addition to a peer-run center including a software program: written material and videos with peers describing their recovery process; reminders of their own actions that give their life meaning and purpose and help to create wellness; a customized survey of symptoms and psychosocial functioning and primary goals for the medication visit; a database; a recovery library; and a one-page report to use in the medication visit.
Horyzons, Australia [65, 84, 85]	Recovery and maintaining clinical benefits	For persons aged 15–25 years with first episode psychosis following discharge from early intervention specialist care in consultation or at home.	A moderated online social networking forum: evaluation of service users’ goals, recovery style, and symptoms; interactive information on psychosis and the recovery process with emphasis on empowerment and social recovery; assessment of strengths; identification of early warning signs and a relapse prevention plan; interactive exercises about activity; cognitive-based strategies; and general overview of the key aspects of the completed modules with an emphasis on personal achievement and recommendation to stay well and to use the social networking features and practice personal strengths.
Miele.Net^a^, Finland [50, 52, 86–89]	To support self-management	For persons aged 18–65 years with schizophrenia spectrum psychoses in inpatient and outpatient settings.	Service user education and online support: information about illness, treatment, well-being, daily activities, and service users’ rights; a channel for peer support; recordings of voices telling service users’ life stories; drawings and pictures; a diary; eSupport; a tool for counseling; and interaction between service users and providers.
ReConnect^b^, Norway [55]	Greater overview and control regarding health and well-being, legitimacy of personal knowledge, strengths and values, and a sense of continuity in relationships	For adult persons with long term mental health problems at different levels of mental health care.	An intervention for guided self-help where service users can: state their values and what is important in their life; describe their current situation, goals and activities related to a wide range of domains; do exercises related to coping strategies, strengths, collaboration with providers, and lifestyle; write a crisis management plan; register information about various aspects of daily life such as sleep, nutrition, physical activity, social life, and medications; get information on issues related to daily life, health, well-being, and social activities including material from peers; register information related to medication; and get access to secure email with their providers.
Wegweis, The Netherlands [51, 56, 64]	Shared decision-making and individual advice related to treatment, rehabilitation, and personal recovery	For persons 18–65 years with schizophrenia or related psychotic disorder for use at home or in a clinical setting.	An Internet-based information and decision tool providing service users: results of their routine outcome monitoring assessments and personalized advice; descriptions of treatment modules dynamically linked to the assessment results; and overview of available treatment modules. Advice is based on evidence-based information, clinical expertise, and service users’ experiences and also refers to the service user’s provider and local counselor.
Your Schizophrenia Care, USA [90]	To empower service users to discuss their mental health treatment with their provider	For adult persons with schizophrenia in outpatient mental health care.	A service user-oriented learning approach related to six areas of quality of care: medications, side effects, referrals, family support, employment, and quality of life. Based on service user’s input on their current status and treatment related to each area, individualized feedback recommendations appear on the screen. The Web site includes video clips designed to model communication strategies and skill, and show how to be proactive in a visit, e.g., by expressing expectations and goals. Service users are encouraged to discuss their responses with their provider in an upcoming visit.

^a^Mieli.Net is called Mental.Net in English

^b^ReConnect was previously called PsyConnect

### How recovery is supported though the Internet interventions

The findings are presented relative to Le Boutillier et al.’s four domains and described with reference to the 16 underlying themes [26].

#### Promoting citizenship

Promoting citizenship domain is characterized by the following themes: seeing beyond the “service user”, service user rights, social inclusion and meaningful occupation [26].

All of the e-recovery interventions in our sample can be said to support this domain and its underlying four themes. The theme of *seeing beyond the “service user”* can be said to be reflected in all the interventions in light of the wide scope and holistic approach that is not limited to diagnosis and symptoms. In addition to peer-support, which in itself may facilitate *social inclusion*, some of the interventions also explicitly offered support for social inclusion such as social networking features and information about service user organizations (Horyzons, Mieli.Net, and ReConnect). Four of the interventions had support for *meaningful occupation* such as daily activities, employment and community activities (Horyzons, Mieli.Net, ReConnect, and Your Schizophrenia Care). Two interventions explicitly provided information and support for *service user rights* (Mieli.Net and ReConnect).

#### Organizational commitment

Organizational commitment is characterized by the following themes: recovery vision, workplace support structures, quality improvement, care pathway and workplace planning [26].

The studies referred to a wide range of concepts that may be construed as a *recovery vision* exemplified by: “a decision support center and computerized tool designed to empower and activate consumers” [49] ”the main principles in the development (…) were patient-centered, health-oriented, supportive self-care abilities and self-management” [50], and; “to empower service users and improve shared decision-making” [51]. Concepts and theories related to recovery such as positive psychology, person-centered care, communication, empowerment, service user involvement, self-management, and shared decision-making were also prominent.

Common for all of the interventions is that they were designed in collaboration with end-users, i.e., both service users and providers, which is an essential part of the theme of *quality improvement*. Justifications offered are mainly to ensure relevance and usefulness. Further, all of the interventions supported service users in becoming more involved in care and as such can be expected to facilitate quality improvement beyond the development phases. *Care pathway* can be interpreted as being reflected in the interventions’ commitment to increase service user access to and participation in the mental health services. Additionally, the interventions enable involvement in recovery-oriented activities outside usual working hours, for example through social support in the evenings and weekends.

*Workplace support structures* and *workplace planning* were not described in the studies.

#### Personally defined recovery

Personally defined recovery is characterized by the following themes: individuality, informed choice, peer support, strengths focus, and holistic approach [26].

The interventions can be said to promote *individuality* or autonomy by explicitly supporting service users in articulating their own values, goals and/or preferences (CommonGround, Horyzons, ReConnect, Wegweis, and Your Schizophrenia Care). Additionally, all of the interventions provided relevant information to promote *informed choice* regarding ongoing care treatment and follow-up. Two of these were designed specifically as shared decision-making tools (CommonGround and Wegweis) while the other four interventions more generally supported service user activation and involvement (Horyzons, Mieli.Net, ReConnect, and Your Schizophrenia Care).

*Peer support* was central to five of the interventions (CommonGround, Horyzons, Mieli.Net, ReConnect and Wegweis). Justifications offered included the role of peer support in promoting learning, self-management and personal responsibility through modeling empowerment and sharing recovery stories as described in the conceptual framework [26]. Peers were also engaged as experts in how to use the intervention (CommonGround, Horyzons, and ReConnect). Experience-sharing through written stories, films and support forums was also an integrated part of the interventions (CommonGround, Mieli.Net, and ReConnect).

Support for the individuals’ own *strengths* was evident in five of the interventions by highlighting what individuals do to stay well (CommonGround), exploring and promoting the individual’s recovery styles (Horyzons), self-care and self-management (Mieli.Net), support in formulating strengths and coping strategies (ReConnect), and advice for personal recovery (Wegweis). All of the interventions have a *holistic approach* in that they include a wide range of domains and themes beyond symptoms such as self-management, strengths, social support, economy, housing and community activities.

#### Working relationship

Working relationship is characterized by the themes partnership and inspiring hope [26].

All of the interventions supported some form of communication between services users and providers such as email, forum, moderation and feedback, which may be said to support aspects of *partnership.* Some of the interventions could be used by the service users from home and/or together with the providers. All of the interventions reflected attitudes towards service users as experts in their own experiences, which is an important part of an inspiring relationship [26]. It was nevertheless not possible to assess the degree to which the communications *inspired hope*.

### Characteristics of the studies

The studies’ place of origin, aims, design, methods, measures and outcomes, and main findings related to each intervention are presented in Table 3 Study characteristics. The number of studies available per interventions varied from one to six.

**Table 3:** Study characteristics

Study	Aim	Methods and outcome measures	Main findings
CommonGround [53]	To describe challenges in decisions about medications and the CommonGround program	Reflections and descriptions based on lived experience and research.	Program being piloted. Promising as support for shared decision-making, decisional conflict and optimizing activities that give life meaning and purpose in recovery.
CommonGround [54]	To describe a 12-month pilot for shared decision-making in psychopharmacology consultation	Observational record for each use of the software from two case management teams for a total of 189 clients. Focus groups with case managers (*n* = 14) medical staff (*n* = 4), clients (*n* = 16) and peer-specialist staff (*n* = 3).	The software was used 662 times varying from one to ten times for each client. Only ten clients refused to use it at some point. The intervention helped practitioners focus on client concerns, enriched dialogue and understanding, made the consultations more effective and empowered client involvement in decision-making.
CommonGround [81]	To describe CommonGround, new role for peers and early adopters, patterns of use and lessons learned	Use of CommonGround database and observation of 8 sites already offering the tool to a total of 4783 users. Log files of use by clients, peers, administrators, therapists, and case managers.	Technology and peer support can enhance shared decision-making even during brief (15-min) psychiatric medication consultations.
CommonGround [49]	Examine the impact of a decision support center and computerized tool on adherence to psychotropic medication	Multivariate linear regression models were used to examine if tool users (*n* = 122) had higher rates of 180-day medication adherence than non-users (*n* = 1000) based on administrative pharmacy claims data.	Relatively good adherence for psychotropic medications at baseline. Intervention had no detectable impact on adherence rates.
CommonGround [82]	To describe the use of self-management strategies, especially a strategy called “Personal Medicine” and how it correlates with wellness and symptom improvement	Pretest-posttest single-group design. A retrospective study of responses from CommonGround health reports generated from 12 clinics and service users (*N* = 5584). A self-developed questionnaire.	Health functioning improved with time. Self-management reduced medication side-effects, increased user satisfaction with medication, and fostered recovery.
CommonGround [83]	Examine the impact of CommonGround on the consumer–doctor interaction in medication consults	Pretest-posttest control group design for four months (*N* = 98). Measure of Patient-Centered Communication (MPCC) and Patient Perception of Patient-Centeredness (PPPC).	No significant effect at baseline. Improved scores after four to six months suggest need for longer intervention and better fidelity.
Horyzons [84]	To describe the rationale and potential of moderated online social therapy and examine the acceptability, safety and initial clinical benefits of system	A six-week trial using an uncontrolled single-group design for participants with first episode psychosis (*N* = 20) participating at least four weeks. Descriptive statistics of log files of use. A questionnaire and semi-structured interviews. Structured Clinical Interview for DSM-IV (SCID patient version), Brief Psychiatric Rating Scale (BPRS), the Calgary Depression Scale for Schizophrenia (CDSS) and the Beck Anxiety Inventory (BAI).	No drop-outs; 70% used the system > three weeks, 95% used social networking, and 60% completed > three modules. The majority of participants reported feeling safe, empowered and more socially connected; 70% considered it a useful post-treatment option. Depressive symptoms were significantly reduced at follow-up.
Horyzons [85]	To assess the safety of Horyzons	Semi structured interviews with participants and simple descriptive statistics from online posts and interviews. Structured Clinical Interview for DSM-IV (SCID patient version) and Brief Psychiatric Rating Scale (BPRS).	No clinical or security problems were noted. Users felt safe using Horyzons.
Horyzons [65]	To determine design guidelines for increasing engagement in mental health applications	Complex health intervention framework in stages: theory, design, exploratory trial, and implementation.	Themes identified for use in guidelines: 1) belonging and security, 2) better understanding of condition, 3) engendering positive thoughts, being accountable and focusing on strengths, and 4) appealing and engaging presentation.
Mieli.Net^a^ [86]	To evaluate usability, quality, functionality, content and benefits of Mieli.Net	Explorative descriptive multiphase study among nurses (*N* = 76), using a questionnaire (The Quality Criteria of Public Online Services) and descriptive statistics, and additionally written feedback and content analysis.	The evaluation showed good portal functionality, relevant content and benefits for users.
Mieli.Net^a^ [52]	Identify barriers and facilitators in implementation of Mieli.Net	Questionnaire after one year use of portal among nurses (*N* = 89). Two thematic open-ended questions analyzed by using content analysis.	Issues emerged in four categories: organization resources, nurses’ individualities, patient-related factors, and portal-related factors. Barriers: lack of computers, time and/or training; nurses’ negative attitudes. Facilitators: easy access to technical resources and Internet; time and motivation among staff.
Mieli.Net^a^ [50]	To describe the design and development process of Mieli.Net	Mixed methods design in four phases: needs analysis (survey for administrative personnel (*n* = 36) and patients (*n* = 316), interviews with patients (*n* = 51) and relatives (*n* = 50) and overview of the literature); development of information areas (relevant literature and feedback from multidisciplinary team and end-users); development of a prototype (information source from 10 focus groups meetings and existing computer-based support systems; and evaluation by clients (*n* = 21), nursing students (*n* = 20) and nurses (*n* = 35).	Five informational areas were identified: illness, treatment, well-being, daily activities, and patient’s rights. Based on this, changes were made in the structure and new applications were added. The service was found to be promising. User involvement in development is important.
Mieli.Net^a^ [87]	To describe nurses’ experiences of information technology-based standardized patient education	Nurses completed a questionnaire about their experiences (*n* = 56), analyzed by content analysis.	The intervention brought the patient and the nurses closer to each other and helped nurses to provide individual support for their patients. The education was time-consuming.
Mieli.Net^a^ [88]	To determine the effectiveness of a needs-based computerized patient education program on patients’ experience of being deprived of their liberty during hospitalization	RCT with three groups: an intervention group with needs based computerized patient education (*n* = 100), a patient education group with conventional education (*n* = 106), and a control group with standard care (*n* = 105). Primary outcome measure patients’ self-reported deprivation of liberty developed for the study, and additionally the PSS-Fin (patient satisfaction scale, Finnish adaptation).	Technology-based education was not found to be superior to other approaches.
Mieli.Net^a^ [89]	To compare user groups’ evaluation of usability	Descriptive design, a small-scale usability study with service users (*n* = 21), nursing students (*n* = 21) and registered nurses (*n* = 35). A self-developed questionnaire about the content, structure and visual appearance of the website.	Service users were positive to intervention tools, but require support. Content, structure and appearance rated as good.
ReConnect^b^ [55]	To describe the design and development process	Community-based participatory research design with particular focus on implications of service user involvement, conducted in two communities by a practice-research-team, involving work-shops with stakeholders and an iterative design process.	The intervention invites a proactive approach from providers. Service users must be involved at all levels of project development to promote person-centeredness.
Wegweis [51]	To investigate the usability of web-based support for routine outcome monitoring (ROM)	A usability study consisting of three types of evaluation; heuristic, qualitative and quantitative with service users (*n* = 15) and ICT experts (*n* = 4). Usability testing grouped into usability topics, verbal reports and observations. Questionnaires .	The prototype has promise for improving ROM practice. Study confirms earlier findings that people with psychotic disorders are not incapable of using web-based systems independently.
Wegweis [64]	To present, evaluate and explain the shared decision support module in Wegweis	An ontology-based approach for selecting and ranking information for service users with schizophrenia based on their routine assessment results. The Manchester short assessment of quality of life (MANSA).	Wegweis pioneers automated interpretation of assessment of results for service users with schizophrenia. Tool interpretations corresponded well with those of clinicians. Service users considered most of the automatically generated advice relevant. The tool is deemed feasible.
Wegweis [56]	To investigate the intervention in naturalistic setting	An open-label, 2-group, parallel RCT with patients with psychosis (*N* = 250) recruited from two outpatient teams, and process evaluation with open interviews with patients (*n* = 15), observation of discussions, and a questionnaire among clinicians. Primary outcome: Combined Outcome Measure for Risk Communication and Treatment Decision-making Effectiveness (COMRADE). Secondary outcome: Client Satisfaction Questionnaire (CSQ).	Inconclusive results on service users’ satisfaction and involvement in decision-making. Poor provider adherence to the protocol may explain the lack of effect. Improved training of clinicians and adaptation of the intervention to the organization is recommended in future studies.
Your Schizophrenia Care [90]	To test the hypothesis that patients assigned to the intervention would be more verbally active and that mental health providers would be more patient-centered in the visit	RCT with patients who have schizophrenia (*N* = 50). The visits were recorded and analyzed using the Roter Interaction Analysis System. Different variables related to communication (e.g., number of statements, patient-centeredness ratio and type of questions asked and information given) and affective tone during visits was measured.	Visits by patients in the intervention group were longer and had greater patient contribution to the dialogue than visit by the control group. Clinicians asked more questions about treatment and the condition than in the control group.

^a^Mieli.Net is called Mental.Net in English

^b^ReConnect was previously called PsyConnect

### Barriers and facilitators

Only Koivunen et al. [52] explicitly addressed barriers and facilitators for implementation as the main study objective, identifying four main categories: organizational resources, nurses’ individual characteristics, patient-related factors, and portal-related factors. Barriers to implementation were lack of computers, lack of time for patients, nurses’ negative attitudes towards computer use and lack of training in how to use the intervention. Technological resources, easy Internet access, time and level of motivation among staff were found to be facilitators of implementation.

Two studies [51, 53] noted problems related to intervention and protocol fidelity which undermined their ability to draw conclusions. They pointed out a need for better training and ensuring the intervention’s optimal fit with the routine practices of participating clinics.

Other reflections included the facilitating role of peers in the use of the intervention [49, 54, 55]. These studies argued that peers not only have more time to give careful guidance, but can sometimes relate more readily to the needs and concerns of service users.

Barriers suggested from the practitioners’ point of view include lack of monetary compensation and a feeling of being overwhelmed [54]. One study noted the concerns of clinicians who feared that if they had access to service-user-controlled content, they could be held responsible for faulty or alarming content [55].

## Discussion

This study offers a first overview of the current state of research at the crossroads of Internet and recovery-oriented practice in conjunction with ordinary care. As such it complements other reviews of technically supported interventions for people with long-term mental health problems related to self-management in general [8–11] and recovery in particular [7, 12].

The e-recovery interventions identified were found to support all four domains in the conceptual framework of recovery-oriented practice [26], reflecting to various degrees 14 of the 16 underlying themes. Overall, the domain supporting personal recovery was most clearly reflected in interventions, whereas the last three domains, i.e., promoting citizenship, organizational commitment and working relationship, were less evident as part of the interventions and accompanying studies. Support for formulation and follow-up of personal goals and preferences, and in accessing peer-support, are key to the personal recovery domain and well supported by the interventions. Also, e-recovery appears to support a broader range of recovery themes than may be feasible in ordinary care, such as social inclusion, meaningful occupation and access to and participation in the care pathway.

The role of peers as an essential element in inspiring hope is fundamental to several of the interventions. However, none of the studies described in detail the hope-inspiring dimension of service user-provider partnerships which is central to recovery [23]. What actually happens during e-mediated interactions, for example, whether interactions between service users and providers are instrumental and detached versus personal and empathetic, and the role of Internet, largely remains a black box [56, 57].

All studies reported service user involvement in intervention design, thus complying with one of the core principles of organizational commitment and the increasing focus on the significance of service user involvement in research to enhance relevance [26, 58, 59]. Two of the 16 themes in the conceptual framework [26] that were not explicitly reflected on by any of the interventions were; workplace support structures and workplace planning. These themes are essential for successful e-recovery implementations and are likely to share challenges that others have noted for e-health in general [60], as well as implementation of recovery-oriented practices per se [28]. Thus, an increased focus on organizational adaptations needed for promoting clinical uses of e-recovery is needed. Interestingly, none of the studies made references to the CHIME framework [38] or the framework of recovery-oriented practice [26], nor reported use of the recommended recovery-specific measures [39–42]. This may be due to the relative recency of the field and the lack of any notable consensus around models and measures. Also, those working with Internet interventions in mental health may find that narrower domains such as shared decision-making and self-management are more readily translatable to an Internet platform than the broader and more multifaceted domain of recovery.

### Dimensions along which Internet can play a transitional role towards recovery-oriented practices

Throughout the analysis of interventions and research, we noted a wide range of impressions and topics that appeared important or relevant in efforts to exploit the potentials of Internet in transitioning towards recovery-oriented practices. Four of these are highlighted in the following.

#### Access to evidence-based interventions

The interventions in our sample build on research supporting isolated components such as shared decision-making and peer support. Due to the multi-component nature of recovery, it will nevertheless remain challenging to identify and compare the active ingredients of e-recovery as a basis for comparing findings. One approach that might aid in doing this is to build on well supported multi-component models (e.g., [29–31, 61]) that are inherently aligned with recovery, but have yet to exploit and assess Internet systematically [62]. The evidence supporting such models might serve as a benchmark for identifying a range of e-recovery and organizational options for improving access to, and/or ways of boosting these models’ active ingredients. Ultimately, it should be possible to incrementally add relevant Internet components in ways that allow assessments of their relative performance. This would also provide opportunities for pursuing new knowledge about recovery.

#### New knowledge about recovery-oriented practice

A fundamental aspect of recovery is that it is *personal* and unique to each individual. Technologies can increasingly ‘learn’ about their users, becoming sensitive and respond to, for example, progress towards one’s personal goals in ways that can increase engagement and reduce attrition [63]. Only one study in our study sample [64] addressed this type of personalization which would seem a key issue for future e-recovery research.

A wide range of theories and tools emerging in technical and social sciences [17–20] and positive computing [21] may aid in examining recovery-relevant issues such as self-esteem, stigma, power, self-disclosure, self-regulation, writing therapy and help-seeking behavior [13–15] and how connecting with others through Internet may enable users to shape the nature of the services in line with recovery principles [17]. However, the opportunities provided by these theories and tools need to be incorporated into studies that specifically address how they may advance recovery processes.

A particularly promising example of an interdisciplinary system design approach that is needed is the study by Lederman et al. [65], the only study in our sample that was published in a technical venue. Their type of approach illustrates our earlier call for joining forces between health service research and technical domains of research in chronic care [66]. By sharing theoretical and methodological rationales for design processes, Lederman and co-workers enable others in the field to build on and compare the various intervention components, and to ultimately illuminate for whom they are effective, under what conditions, and why.

#### Values-based research and practices

Empowering technologies [13, 66] can also be disempowering [67, 68] depending on whose values and conceptions of evidence inform decisions about uses of new technologies on individual, professional, and societal levels [69]. While it may be easy to dismiss on moral grounds innovations like ‘chip in a pill’ (e.g., wireless surveillance of medication consumption via biological markers) that warns clinicians of a patient’s poor compliance [70], vigilance is also necessary for less obvious pitfalls. For example, an e-recovery tool that encourages service users to formulate and share with providers what is important to them, along with their personal goals, creates an obligation for providers to respond by seriously exploring realistic ways of adapting services to facilitate those goals. Arguably, if service providers are not prepared to do this, then offering such a tool would be ethically questionable.

The ethical issues associated with ubiquitous e-health-systems need constant attention, and they need it urgently [71]. Slade [23] argues that an important first step in all service design and daily clinical decision-making is to make the values of those involved explicit, and hence amenable to debate. Giving primacy to patient autonomy over biomedical beneficence appears as a defining value in recovery-oriented practices [23]. Interestingly, the epidemic growth of multiple long-term conditions in aging populations has prompted the biomedical literature to revisit similar ethical premises in efforts to resolve conflicting health and professional goals and their underlying values [72, 73]. Arguably, chronic care and recovery researchers could benefit from joining forces towards a values-based, alternative health outcome paradigm [72], accompanying measures [39–41] and required changes to support systems [74, 75]. Finally, EU’s Responsible Research and Innovation (RRI) mission and participatory methods are well aligned with the values of recovery and can be an important ally in promoting an ethically sound e-recovery agenda.

#### Internet as a facilitator for organizational transformation

Implementing organizational transformation towards recovery-oriented services has been found to be challenging [32–35]. It is probably unlikely that e-recovery interventions will demonstrate recovery-oriented findings, as long as they operate within organizations that work at odds with recovery principles. Also, barriers to electronic communication in clinical environments [76], along with clinician resistance and uncertainty towards transformation of therapeutic relationships in line with partnership principles [77], are likely to impede e-recovery practices. Nevertheless, incremental changes currently facilitated by narrow components of e-recovery, e.g., shared decision-making [78], may stimulate new types of dialogue and insights that in turn stimulate broader organizational changes aligned with recovery.

### Strengths and limitations

This study shares the strengths and limitations of scoping studies in that it illuminates the volume, nature and characteristics of the field of interest, but does not appraise the quality or weight of evidence [43]. A strength is use of the conceptual framework for recovery-oriented practice as a basis for analysis and description of findings. This can turn aid in refining concepts during this formative stage of development, along with enabling a proactive and systematic discourse about Internet-based recovery-oriented interventions. However, limiting the inclusion criteria to interventions used in conjunction with ordinary care led to exclusion of interventions with potentially important contributions to the emerging field of e-recovery (e.g., [79, 80]). This can also be said of our exclusion of gray literature. Thus, we cannot claim to represent all the current work that is relevant to advancing e-recovery.

## Conclusion

The e-recovery interventions and research identified in this review are breaking new ground in an area that can be expected to expand. The degree to which e-recovery is contributing to the cultural and organizational changes called for in reorienting mental health care towards recovery practices cannot be deciphered from our study [25]. Nevertheless, incremental adaptations of components that can potentially facilitate recovery-oriented care were clearly evident in our study [77]. Technologies that may potentially aid in illuminating and facilitating recovery processes are still in their formative stages. We suggest a preliminary road-map for an e-recovery research and innovation agenda attending to four dimensions: access to evidence-supported interventions, new knowledge about personal recovery, values-based approaches, and Internet as a facilitator for organizational transformation. Recovery-oriented researchers and practitioners need to exploit the potentials of Internet in shaping interventions that ultimately promote recovery among those in need of long term mental health support.

## Abbreviations

CHIME: Connectedness to others and the community; hope and optimism about the future; identity building beyond being a patient and towards a positive sense of identity without stigma; meaning inlife; and empowerment
e-recovery interventions: Internet-based interventions that support the four practice domains described in the framework for recovery-oriented practice: personal recovery, promoting citizenship, organizational commitment and working relationship
Recovery: A way of living a satisfying, hopeful, and contributing life even with any limitations caused by illness

## References

[1] Reynolds J, Griffiths KM, Cunningham JA, Bennett K, Bennett A (2015). Clinical practice models for the use of E-mental health resources in primary health care by health professionals and peer workers: a conceptual framework. JMIR Mental Health.

[2] Andersson G, Titov N (2014). Advantages and limitations of Internet-based interventions for common mental disorders. World Psychiatry.

[3] Lal S, Adair CE (2014). E-mental health: a rapid review of the literature. Psychiatr Serv.

[4] Coull G, Morris PG (2011). The clinical effectiveness of CBT-based guided self-help interventions for anxiety and depressive disorders: a systematic review. Psychol Med.

[5] Barak A, Hen L, Boniel-Nissim M, Shapira N (2008). A comprehensive review and a meta-analysis of the effectiveness of internet-based psychotherapeutic inteventions. J Technol Hum Serv.

[6] Andersson G, Cuijpers P (2009). Internet-based and other computerized psychological treatments for adult depression: a meta-analysis. Cogn Behav Ther.

[7] Leitan ND, Michalak EE, Berk L, Berk M, Murray G. Optimizing delivery of recovery-oriented online self-management strategies for bipolar disorder: a review. Bipolar Disord. 2014;17:115–127. 10.1111/bdi.12258 25238632

[8] van der Krieke L, Wunderink L, Emerencia AC, de Jonge P, Sytema S (2014). E-mental health self-management for psychotic disorders: state of the art and future perspectives. Psychiatr Serv.

[9] Barlow JH, Ellard DR, Hainsworth JM, Jones FR, Fisher A (2005). A review of self-management interventions for panic disorders, phobias and obsessive-compulsive disorders. Acta Psychiatr Scand.

[10] Murray E (2012). Web-based interventions for behavior change and self-management: potential, pitfalls, and progress. Med 2 0.

[11] Karasouli E, Adams A (2014). Assessing the evidence for e-resources for mental health self-management: a systematic literature review. JMIR Mental Health.

[12] Naslund JA, Marsch LA, McHugo GJ, Bartels SJ. Emerging mHealth and eHealth interventions for serious mental illness: a review of the literature. J Ment Health. 2015;24:320–331. 10.3109/09638237.2015.1019054 PMC4924808 26017625

[13] Amichai-Hamburger Y, McKenna KY, Tal S-A (2008). E-empowerment: empowerment by the internet. Comput Human Behav.

[14] Johnsen J-AK, Gammon D. Connecting with ourselves and others online: psychological aspects of online health communication. Patient-Centered E-Health New York, NY: Medical Information Science Reference. 2009:26–46.

[15] Bjerkan J, Vatne S, Hollingen A (2014). Web-based collaboration in individual care planning challenges the user and the provider roles - toward a power transition in caring relationships. J Multidiscip Healthc.

[16] Dixon LB, Holoshitz Y, Nossel I (2016). Treatment engagement of individuals experiencing mental illness: review and update. World Psychiatry.

[17] Alvarez-Jimenez M, Gleeson JF, Bendall S, Lederman R, Wadley G, Killackey E (2012). Internet-based interventions for psychosis: a sneak-peek into the future. Psychiatr Clin North Am.

[18] Gaggioli A, Pioggia G, Tartarisco G, Baldus G, Corda D, Cipresso P (2013). A mobile data collection platform for mental health research. Pers Ubiquit Comput.

[19] Yuen EK, Herbert JD, Forman EM, Goetter EM, Comer R, Bradley J-C (2013). Treatment of social anxiety disorder using online virtual environments in second life. Behav Ther.

[20] Cugelman B (2013). Gamification: what it is and why it matters to digital health behavior change developers. JMIR Serious Games.

[21] Calvo RA, Peters D. Positive Computing: Technology for wellbeing and human potential. Cambridge: MIT Press; 2014.

[22] Anthony WA (1993). Recovery from mental illness: the guiding vision of the mental health service system in the 1990s. Psychosoc Rehabil J.

[23] Slade M (2009). Personal recovery and mental illness: a guide for mental health professionals.

[24] Davidson L, Roe D (2007). Recovery from versus recovery in serious mental illness: one strategy for lessening confusion plaguing recovery. J Ment Health.

[25] Davidson L, Rowe M, Tondora J, O’Connell MJ, Lawless MS. A practical guide to recovery-oriented practice: Tools for transforming mental health care. Oxford: Oxford University Press; 2008.

[26] Le Boutillier C, Leamy M, Bird VJ, Davidson L, Williams J, Slade M (2011). What does recovery mean in practice? A qualitative analysis of international recovery-oriented practice guidance. Psychiatr Serv.

[27] Graf TR, Bloom FJ, Tomcavage J, Davis DE (2012). Value-based reengineering: twenty-first century chronic care models. Prim Care.

[28] van der Krieke L, Bird V, Leamy M, Bacon F, Dunn R, Pesola F (2015). The feasibility of implementing recovery, psychosocial and pharmacological interventions for psychosis: comparison study. Implement Sci.

[29] Cook JA, Jonikas JA, Hamilton MM, Goldrick V, Steigman PJ, Grey DD (2013). Impact of wellness recovery action planning on service utilization and need in a randomized controlled trial. Psychiatr Rehabil J.

[30] Fardig R, Lewander T, Melin L, Folke F, Fredriksson A (2011). A randomized controlled trial of the illness management and recovery program for persons with schizophrenia. Psychiatr Serv.

[31] Drake RE, Bond GR, Becker DR. Individual placement and support: an evidence-based approach to supported employment. Oxford: Oxford University Press; 2012.

[32] Slade M, Amering M, Farkas M, Hamilton B, O’Hagan M, Panther G (2014). Uses and abuses of recovery: implementing recovery-oriented practices in mental health systems. World Psychiatry.

[33] Perkins R, Slade M (2012). Recovery in England: transforming statutory services?. Int Rev Psychiatry.

[34] Oades LG, Anderson J (2012). Recovery in Australia: Marshalling strengths and living values. Int Rev Psychiatry.

[35] Vandekinderen C, Roets G, Roose R, Van Hove G (2012). Rediscovering recovery: reconceptualizing underlying assumptions of citizenship and interrelated notions of care and support. Sci World J.

[36] Slade M (2010). Mental illness and well-being: the central importance of positive psychology and recovery approaches. BMC Health Serv Res.

[37] Mueser KT, Corrigan PW, Hilton DW, Tanzman B, Schaub A, Gingerich S (2002). Illness management and recovery: a review of the research. Psychiatr Serv.

[38] Leamy M, Bird V, Le Boutillier C, Williams J, Slade M (2011). Conceptual framework for personal recovery in mental health: systematic review and narrative synthesis. Br J Psychiatry.

[39] Sklar M, Groessl EJ, O’Connell M, Davidson L, Aarons GA (2013). Instruments for measuring mental health recovery: a systematic review. Clin Psychol Rev.

[40] Shanks V, Williams J, Leamy M, Bird VJ, Le Boutillier C, Slade M (2013). Measures of personal recovery: a systematic review. Psychiatr Serv.

[41] Burgess P, Pirkis J, Coombs T, Rosen A (2010). Review of recovery measures. Australian mental health outcomes and classification network.

[42] Macpherson R, Pesola F, Leamy M, Bird V, Le Boutillier C, Williams J, et al. The relationship between clinical and recovery dimensions of outcome in mental health. Schizophr Res. 2015;175:142–7. 10.1016/j.schres.2015.10.031 26527245

[43] Arksey H, O’Malley L (2005). Scoping studies: towards a methodological framework. Int J Soc Res Methodol.

[44] Colquhoun HL, Levac D, O’Brien KK, Straus S, Tricco AC, Perrier L, et al. Scoping reviews: time for clarity in definition, methods, and reporting. J Clin Epidemiol. 2014;67:1291–4. 10.1016/j.jclinepi.2014.03.013 25034198

[45] Levac D, Colquhoun H, O’Brien KK (2010). Scoping studies: advancing the methodology. Implement Sci.

[46] Peters MD, Godfrey CM, Khalil H, McInerney P, Parker D, Soares CB (2015). Guidance for conducting systematic scoping reviews. Int J Evid Based Healthc.

[47] Braun V, Clarke V (2006). Using thematic analysis in psychology. Qual Res Psychol.

[48] Graneheim UH, Lundman B (2004). Qualitative content analysis in nursing research: concepts, procedures and measures to achieve trustworthiness. Nurse Educ Today.

[49] Stein BD, Kogan JN, Mihalyo MJ, Schuster J, Deegan PE, Sorbero MJ (2013). Use of a computerized medication shared decision making tool in community mental health settings: impact on psychotropic medication adherence. Community Ment Health J.

[50] Välimäki M, Anttila M, Hätönen H, Koivunen M, Jakobsson T, Pitkänen A (2008). Design and development process of patient-centered computer-based support system for patients with schizophrenia spectrum psychosis. Inform Health Soc Care.

[51] van der Krieke L, Emerencia AC, Aiello M, Sytema S (2012). Usability evaluation of a web-based support system for people with a schizophrenia diagnosis. J Med Internet Res.

[52] Koivunen M, Hatonen H, Valimaki M (2008). Barriers and facilitators influencing the implementation of an interactive Internet-portal application for patient education in psychiatric hospitals. Patient Educ Couns.

[53] Deegan PE (2007). The lived experience of using psychiatric medication in the recovery process and a shared decision-making program to support it. Psychiatr Rehabil J.

[54] Deegan PE, Rapp C, Holter M, Riefer M (2008). Best practices: a program to support shared decision making in an outpatient psychiatric medication clinic. Psychiatr Serv.

[55] Gammon D, Strand M, Eng LS (2014). Service users’ perspectives in the design of an online tool for assisted self-help in mental health: a case study of implications. Int J Ment Health Syst.

[56] van der Krieke L, Emerencia AC, Boonstra N, Wunderink L, de Jonge P, Sytema S (2013). A web-based tool to support shared decision making for people with a psychotic disorder: randomized controlled trial and process evaluation. J Med Internet Res.

[57] Sucala M, Schnur JB, Constantino MJ, Miller SJ, Brackman EH, Montgomery GH (2012). The therapeutic relationship in e-therapy for mental health: a systematic review. J Med Internet Res.

[58] Stewart R, Liabo K (2012). Involvement in research without compromising research quality. J Health Serv Res Policy.

[59] Brett J, Staniszewska S, Mockford C, Herron-Marx S, Hughes J, Tysall C (2014). A systematic review of the impact of patient and public involvement on service users, researchers and communities. Patient.

[60] Greenhalgh T, Robert G, Macfarlane F, Bate P, Kyriakidou O (2004). Diffusion of innovations in service organizations: systematic review and recommendations. Milbank Q.

[61] Bouvet C, Battin C, Le Roy-Hatala C (2015). The clubhouse model for people with severe mental illnesses: literature review and French experiment. Encéphale.

[62] Lord SE, McGurk SR, Nicholson J, Carpenter-Song EA, Tauscher JS, Becker DR (2014). The potential of technology for enhancing individual placement and support supported employment. Psychiatr Rehabil J.

[63] Graffigna G, Barello S, Wiederhold BK, Bosio AC, Riva G (2013). Positive technology as a driver for health engagement. Stud Health Technol Inform.

[64] Emerencia A, van der Krieke L, Sytema S, Petkov N, Aiello M (2013). Generating personalized advice for schizophrenia patients. Artif Intell Med.

[65] Lederman R, Wadley G, Gleeson J, Bendall S, Alvarez-Jiménez M (2014). Moderated online social therapy: designing and evaluating technology for mental health. ACM Trans Comput Hum Interaction (TOCHI).

[66] Gammon D, Berntsen GK (2015). The chronic care model and technological research and innovation: a scoping review at the crossroads. J Med Internet Res.

[67] Hacker KL, Morgan EL (2013). Empowering and disempowering aspects of new media networking and digital democracy. Int J Technol Diff (IJTD).

[68] Gammon D, Christiansen E, Wynn R (2009). Exploring morally relevant issues facing families in their decisions to monitor the health-related behaviours of loved ones. J Med Ethics.

[69] Lopes E, Carter D, Street J (2015). Power relations and contrasting conceptions of evidence in patient-involvement processes used to inform health funding decisions in Australia. Soc Sci Med.

[70] Medication Mechanization: Microchip Sensors in Abilify to Increase Medication Compliance. http://www.madinamerica.com/. Accessed 16 Jan 2016.

[71] Lupton D (2012). M-health and health promotion: the digital cyborg and surveillance society. Soc Theory Health.

[72] Reuben DB, Tinetti ME (2012). Goal-oriented patient care—an alternative health outcomes paradigm. N Engl J Med.

[73] Berntsen G, Gammon D, Steinsbekk A, Salamonsen A, Foss N, Ruland C (2015). How do we deal with multiple goals for care within an individual patient trajectory? A document content analysis of health service research papers on goals for care. BMJ Open.

[74] Kim K, Nahm E (2012). Benefits of and barriers to the use of personal health records (PHR) for health management among adults. Online J Nurs Informatics.

[75] Dullabh PM, Sondheimer NK, Katsh E, Evans MA (2014). How patients can improve the accuracy of their medical records. EGEMS.

[76] Varsi C, Ekstedt M, Gammon D, Ruland CM (2015). Using the consolidated framework for implementation research to identify barriers and facilitators for the implementation of an internet-based patient-provider communication service in five settings: a qualitative study. J Med Internet Res.

[77] Park MM, Lencucha R, Mattingly C, Zafran H, Kirmayer LJ (2015). A qualitative study on the ethics of transforming care: examining the development and implementation of Canada’s first mental health strategy. Implement Sci.

[78] Perestelo-Perez L, Gonzalez-Lorenzo M, Perez-Ramos J, Rivero-Santana A, Serrano-Aguilar P (2011). Patient involvement and shared decision-making in mental health care. Curr Clin Pharmacol.

[79] Simon GE, Ludman EJ, Goodale LC, Dykstra DM, Stone E, Cutsogeorge D (2011). An online recovery plan program: can peer coaching increase participation?. Psychiatr Serv.

[80] Todd NJ, Jones SH, Hart A, Lobban FA (2014). A web-based self-management intervention for Bipolar Disorder ‘living with bipolar’: a feasibility randomised controlled trial. J Affect Disord.

[81] Deegan PE (2010). A web application to support recovery and shared decision making in psychiatric medication clinics. Psychiatr Rehabil J.

[82] MacDonald-Wilson KL, Deegan PE, Hutchison SL, Parrotta N, Schuster JM (2013). Integrating personal medicine into service delivery: empowering people in recovery. Psychiatr Rehabil J.

[83] Campbell SR, Holter MC, Manthey TJ, Rapp CA (2014). The effect of CommonGround software and decision support center. Am J Psychiatr Rehabil.

[84] Alvarez-Jimenez M, Bendall S, Lederman R, Wadley G, Chinnery G, Vargas S (2013). On the HORYZON: moderated online social therapy for long-term recovery in first episode psychosis. Schizophr Res.

[85] Gleeson JF, Lederman R, Wadley G, Bendall S, McGorry PD, Alvarez-Jimenez M (2014). Safety and privacy outcomes from a moderated online social therapy for young people with first-episode psychosis. Psychiatr Serv.

[86] Koivunen M, Valimaki M, Pitkanen A, Kuosmanen L (2007). A preliminary usability evaluation of Web-based portal application for patients with schizophrenia. J Psychiatr Ment Health Nurs.

[87] Anttila M, Koivunen M, Valimaki M (2008). Information technology-based standardized patient education in psychiatric inpatient care. J Adv Nurs.

[88] Kuosmanen L, Valimaki M, Joffe G, Pitkanen A, Hatonen H, Patel A (2009). The effectiveness of technology-based patient education on self-reported deprivation of liberty among people with severe mental illness: a randomized controlled trial. Nord J Psychiatry.

[89] Kuosmanen L, Jakobsson T, Hyttinen J, Koivunen M, Valimaki M (2010). Usability evaluation of a web-based patient information system for individuals with severe mental health problems. J Adv Nurs.

[90] Steinwachs DM, Roter DL, Skinner EA, Lehman AF, Fahey M, Cullen B (2011). A web-based program to empower patients who have schizophrenia to discuss quality of care with mental health providers. Psychiatr Serv.

